# COVID-19 Unintended Effects on Breast Cancer in Italy After the Great Lockdown

**DOI:** 10.3389/fpubh.2020.601748

**Published:** 2020-12-16

**Authors:** Chiara Oldani, Gianluca Vanni, Oreste Claudio Buonomo

**Affiliations:** ^1^Department of Economics and Engineering, University of Viterbo “La Tuscia”, Viterbo, Italy; ^2^Breast Unit, Policlinico Tor Vergata University, Rome, Italy

**Keywords:** breast cancer, screening, COVID-19, patients' sensibilization, Italy, European recovery plan, public health, global policy

## Abstract

Italy introduced social distancing measures, which limited the spread of COVID-19; all the non-life-threatening treatments have been temporarily suspended, including screening programs. This decision leads to unintended effects on the ability to detected neoplasia in their first stages. Possible future outcomes of the ability to detect new breast cancer cases based on two alternative scenarios show that the reduction in organized screening activities will limit the ability to detect no <3.43% of the new cases; the economic crisis will reduce voluntary screening, increasing the undetected new cases up to 11.73%. Cases diagnosed with delay will show up in their advanced stage along with unknown effects on mortality and health care costs. Global health care policies should be implemented to counterbalance these adverse effects.

## Introduction

On 31 January 2020 the spread of the New Coronavirus SARS2—named COVID-19 has been officially announced by the World Health Organization (1). The pandemic has been declared in March 2020 and the state of emergency will last until January 2021 in Italy. Different epidemiological models provide slightly different projections over the period necessary for the reproduction number (R0) of the COVID-19 virus to fall below (1): this condition confirm that emergency are apparently under control. The Imperial College (2) model estimates that the minimum period necessary to stop the spreading of the COVID-19 is 12–15 weeks (i.e., 3–4 months); presence of COVID-19 virus has been detected in November in China, and in late December first case was reported in North of Italy. China has been the first to lockdown the country in January 2020 to limit the exponential spread of the reproduction number of the virus COVID-19, Italy followed in March 2020, the first country in Europe and among the G-7. During the lockdown period, social distancing measures have been introduced (3, 4). “Global health security is a shared responsibility; it requires a collaborative collective response based on transparency and trust (5).” Sanitary uncertainty due to COVID-19 revealed several systemic weaknesses and has been translated into economic effects that are similar to those of a war; according to available forecasts (6), the COVID-19 pandemic will create a structural break in the public expenditure, namely of health care, social expenditure and unemployment benefits, and ultimately on public debts. Most European countries have explicitly adopted principles of rights and duties to address the COVID-19 health emergency. The Prime ministers of France, Italy, Spain, and Germany in March and April 2020 have all publicly declared that “cost” will not be a consideration in fighting the COVID-19 virus, or in making medical treatment available; the analytical framework adopted by these governments accepts a fiduciary duty to protect their citizens' health. On the other side, countries as Sweden and Brazil, that adopted the cost-benefit analysis to manage the health emergency; their choice not to lock down the country has been insensitive to both distributive and rights-based considerations (7). The medium to long-term effects of the pandemic depend on various known and unknown factors, and the economic literature provides little help to guide for policymakers. Available forecasts consider:

1. The duration of the lockdown and the (estimated) number of spikes in the curve of infected-sick in 2020–2021.
2. Direct effects on consumption, investment, unemployment, mortality, and public health care expenditure rates.
3. Side effects (including psychological) on the economy and population; some (but not all) are the growing lack of confidence, social stigma for sick persons-population, depression, and anxiety.

The social distancing measures have involved virtually all sectors, from tourism to restaurant, beauty salons, hairdressers, and even the public and private health systems. During the lockdown, the health system has been turned upside-down. Most of the healthcare resources have been shifted toward COVID-19 patients at the expense of other patients deemed non-urgent. This resource reallocation, in addition to COVID-19 cross infection risk and patients' anxiety of the virus (8), lead the Italian National Health Service (NHS) to provide only urgent procedures. During the lockdown period, all the non-life-threatening treatments have been temporarily suspended, including screening programs (9). This emergency decision probably did not consider the possible unintended consequences.

Millions of citizens take advantage of screening, allowing early diagnosis. Among the different programs, one of the most popular is that of breast cancer screening (10).

**Figure 1:**
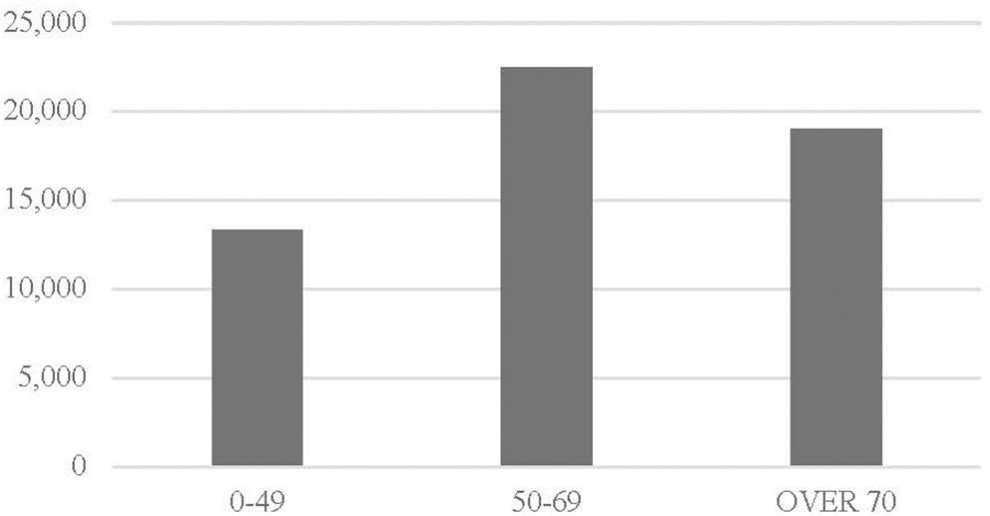
Italian women: breast cancer by age in 2017. Source: Eurostat (11).

Breast cancer is a socially relevant disease. It is the most common cause of cancer death in the European Union which was 7% in 2016, according to Eurostat (11); in 2016, breast cancer caused 97,000 deaths in Europe and 12,000 in Italy. Screening activities have substantial positive effects in terms of reduced incidence of advanced breast cancer, loco regional recurrence and mortality along with a reduction of health care costs, measured by hospital stay, need for chemotherapy and invasive treatments (12, 13). Breast cancer is the most diagnosed oncological disease in women, involving in Italy more than 50,000 women every year (Figure 1). Research has largely contributed to the reduction in perspective mortality caused by breast cancer. Despite high incidence rate, during the last years there has been a substantial improvement in terms of oncological outcome with a survival rate of 87% at 5 years (14). The improvement was associated both with evolutions of treatments and earlier diagnosis due to screening (15). Breast cancer screening was introduced in Italy in the second half of the 1990s, provided to women aged 50–69 with a mammography every 2 years.

The NHS in G-7 countries devoted substantial resources to strengthen these programs also through patients' sensibilization to periodical controls (16). Over the last 30 years the number of screening activities has increased, and in 2017 (17) the Italian NHS provided over 4.5 million organized screenings that benefited 54.6% of women aged 50–69, while over 1.6 million women accessed voluntary screening, covering another 19.23% of women aged 50–69. Following screening activities organized by the Italian NHS, 8,257 neoplasia cases have been detected, 37% of the total (22,482). Despite over-diagnosis and overtreatment risk, screening (organized and voluntary) can diagnose ~65% of breast cancers with negative features at clinical examination (13).

In this paper, we focus on the indirect effects of the breast cancer screening suspension. Due to temporary suspension of breast cancer screening, we will probably observe an increase in advanced breast cancer diagnosis, with a corresponding deterioration of the quality of life and oncological outcome for breast cancer patients, accompanied by an increase in health care costs. We aim to provide the scientific community with a forecast of breast cancer undetected cases, by considering two different scenarios. The Italian experience can help other countries that introduced social distancing measures to implement public health care policies within the NHS to counterbalance these adverse effects.

## Methods

Scenario analysis represents a sequence of hypothetical events with the purpose of focusing on causal points. Scenario analysis can describe possible future outcomes of the present social distancing policies. The baseline is the last year available, 2017; based on Italian female population data (17), screening performed and on the ability of screening to detect neoplasia, we forecast the undetected cases, as a consequence of the reduction of screening (18). Each month of screening suspension, ceteris paribus, leads to 1/12 reduction of screening activities and to a proportional reduction of neoplasia detection (19). We consider two alternative scenarios; the hypothesis of scenario 1 (optimistic 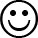) states that organized screening activities missed during the lockdown months are not performed in the remaining months of 2020 (light blue bar), if compared with the baseline year, while voluntary screening activities (gray bar) are performed.

The hypotheses of scenario 2 (pessimistic 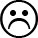) are that missed organized screening activities during the lockdown months are not performed in the remaining months of 2020 (light blue bar), as well as voluntary screening activities (gray bar) if compared with the baseline year; the latter diminishes due to the significant reduction in disposable income of all households that minimize the expenses on non-urgent health care, including voluntary screening.

## Results

**Figure 2:**
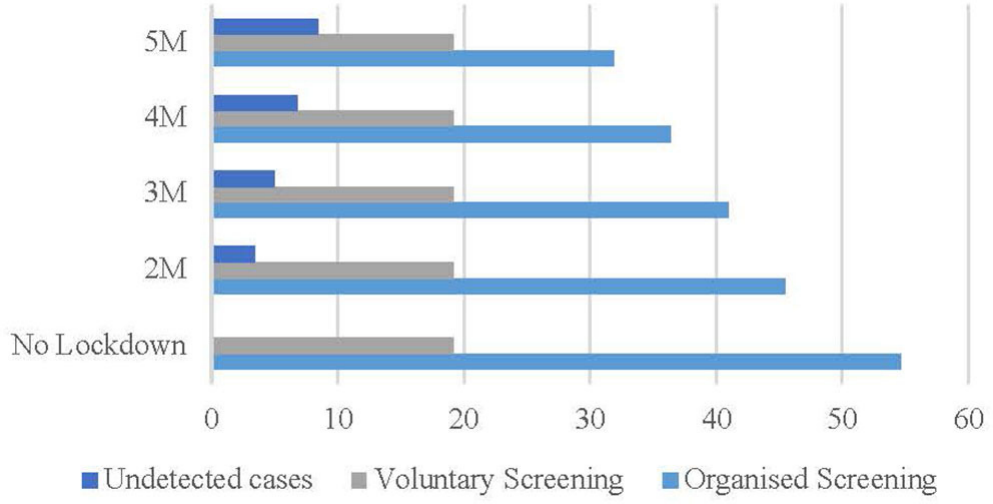
Optimistic scenario (%). Source: Osservatorio Screening (17) and authors' elaboration.

In the optimistic scenario (Figure 2), if the restriction on non-urgent activities lasts from the beginning of March to the beginning of May 2020, 3.43% of cases (blue bar) will not be detected. Considering longer periods, 5.01% (3 months), 6.77% (4 months), and 8.42% (5 months) of cases (gray bar) will not be detected.

The optimistic scenario is coherent with the fact that the (higher) level of income and education positively correlate with (more) voluntary screening activities (19, 20) estimated in regard to the US labor market that job and income losses due to the COVID-19 pandemic have been smaller among workers with higher level of education.

**Figure 3:**
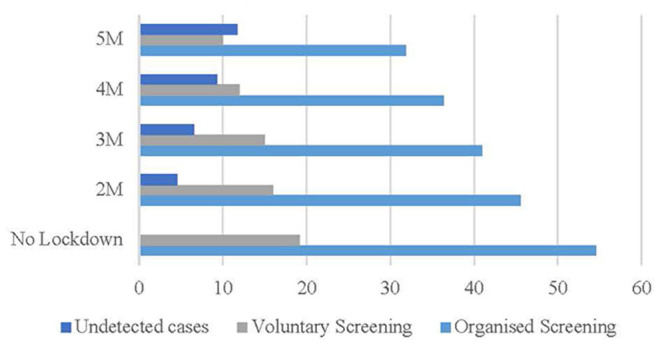
Pessimistic scenario (%). Source: Osservatorio Screening (17) and authors' elaboration.

In the pessimistic scenario (Figure 3), if the restriction on non-urgent activities lasts from the beginning of March to the beginning of May 2020, 4.53% of cases (blue bar) will not be detected. Considering longer lockdown periods, 6.57% (3 months), 9.34% (4 months), and 11.73% (5 months) of cases (gray bar) will not be detected.

The scenario is coherent with the fact that in OECD countries women are more likely to be in temporary, part-time, and precarious employment (21). In Italy, the gender gap in the labor market is larger than 20 percentage points and the pandemic will increase the burden of home and childcare on women, due to shut down of schools and kinder-gardens.

## Discussion of Results

The health care lockdown in Italy has not been shorter than 4 months; only in the summer of 2020, the Italian Government has intervened to remove the suspension of all the non-life-threatening treatments, including cancer screening. Time has key role to reduce the unintended consequences of the pandemic; the longer the health care lockdown lasts, the higher will be the final effects in terms of morbidity, mortality, and health care costs.

Many studies have attempted to estimate breast cancer growth time. Data reported in the literature estimate doubling tumor times varying from 42 to 260 days. This poor accuracy measurement, correlated with the different biological characteristics of breast tumors, is unhelpful for determining the effect of delays on the clinical presentation of breast cancer. However, we can reasonably suppose, based on (22, 23), that in 6 months up to 50% of breast cancer cases could increase tumor dimension in up to 1 cm.

After the introduction of breast cancer screening programs, we have detected a turnaround in breast cancer clinical presentation: a reduction of palpable lesions (local advance breast cancer) and an increase of un-palpable lesions (early stage) (24). Due to the temporary suspension of the screening during the lockdown, we have already observed a reduction of breast cancer diagnosis cases (9). The diagnoses that are performed during the lockdown period are of clinically evident lesions (palpable lesions, nipple discharge, cutis retraction, breast ulceration, and mastitis carcinomatosa) which correspond to about 35–40% of all breast cancer lesions (24). The reduction of breast cancer diagnoses would lead to an increase in new cases once the lockdown period ends, an increase that could undermine the cancer health system which is already experiencing a significant slowdown with a consequent growth of waiting lists (25).

Interruption and partial reduction of the public and private breast cancer screening activities can lead to detecting new cases of BC in advanced stage. Failure to early diagnoses could lead to an increase of more invasive surgery, need for further treatments such as systemic chemotherapy impacting women's quality of life, worst oncological outcomes and increased NHS costs. Data reported on US commercially insured population (22) between 2009 and 2012 (n. 8,360) showed that the costs of treating breast cancer could be reduced achieving early diagnoses and treatments: “*earlier detection of breast cancer by routine screening leads not only to reduced morbidity and mortality but also to lower costs for cancer treatment”* (p. 31). Similar investigations are not available for the Italian population. However, in Europe treatment costs are higher for patients with advanced breast cancer.

## Conclusion and Policy Implications

Regardless of country specific strategy to manage COVID-19 pandemic, the ability to look at the consequences of state actions beyond the remit of the current health emergency is crucial in the wider context of global policy-making (26). Key points in public health policy response are funding and patients' sensibilization.

About funding, the European recovery plan settled by the Eurogroup (27) will provide countries with liquidity, funding and flexibility on current budget rules. The EU is moving faster than we are accustomed to, and history teaches us that policy coordination is the only successful exit strategy following a systemic shock, like the COVID-19. The €6 billion Health Initiative launched by the European Commission (28) is only a starting point in the management of the emergency. Further coordinate response and funding are needed to tackle the direct and indirect consequences of the pandemic, and in particular to fill the gap in cancer screening. The size of the health care funding necessary in Italy, similarly to other European countries, depends on its medium and long-term objectives. Organized cancer screening should be considered firstly of health care managers due to it is a cost-effective mean to reduce health care costs and mortality.

About patients' sensibilization, it is very likely that screening adhesion by patients will be lower than in the pre-COVID-19 era. This is supported by three main reasons. Firstly, the Italian healthcare system may not be able to fill in the gap due to the restriction on non-urgent activities and meet the patients' demand for mammography, especially in the center and south of the country. Secondly, the social distancing measures have a substantial impact on women's income that in turn could lead to a reduction in medical expenditure (i.e., reduction of voluntary screening). The gender gap in the Italian labor market is likely to increase further following the pandemic, especially for low-skilled and uneducated women, thus the risk of poverty (20, 21). Thirdly, patients' anxiety should not be underestimated. During the lockdown, patients with breast cancer diagnosis often refused to undergo surgery due to the COVID-19 anxiety (8, 9, 24). Therefore, a portion of women may choose not to adhere to screening campaigns in the coming months of 2020 (29).

Socio-economic and health consequences of results are relevant in both scenarios under consideration. In the optimistic scenario the undetected cases rate ranges between 5 and 8.42%. Otherwise, in the pessimistic scenario undetected cases rate raise from 6.57 to 11.53%. Impairment of early tumor detection could result in higher health care cost and worsening of long-term outcome. In our opinion, at the end of the pandemic, health care policies should be implemented within the NHS to counterbalance these unintended effects. To fill in the gap and meet patients' demands, it is necessary to re-finance regular screening within the Italian NHS. Voluntary screening should also be favored with national targeted media campaigns on newspapers, social media, radio, and the TV.

First COVID-19 outbreak was greatly overcome by Italian NHS, but cross-infection within hospital between health care workers and patients generates anxiety among workers and patients (8, 29). A nationwide informative campaign on the procedures to manage the risks of COVID-19 within the health care system and their effects could help to reduce patients' anxiety; tradition and social media campaigns, together with contact-tracing apps, like the Italian Immuni, can be employed to share relevant information among sensible citizens.

## Author Contributions

CO: conceptualization, data, methodology, writing original draft preparation, and writing- reviewing. GV: conceptualization, data, and writing original draft preparation. OB: supervision and writing- reviewing and editing. All authors contributed to the article and approved the submitted version.

## Conflict of Interest

The authors declare that the research was conducted in the absence of any commercial or financial relationships that could be construed as a potential conflict of interest.

## References

[1] World Health Organization Coronavirus Disease COVID-19 Pandemic (2020). Available online at: https://www.who.int/emergencies/diseases/novel-coronavirus-2019 (accessed May 22, 2020).

[2] MRC Centre for Global Infectious Disease Analysis COVID-19 Report 13 (2020). London Available online at: https://www.imperial.ac.uk/mrc-global-infectious-disease-analysis/covid-19/ (accessed May 22, 2020).

[3] Italian Prime Minister. Misure Urgenti in Materia di Contenimento e Gestione Dell'emergenza Epidemiologica da COVID-19 - 9 March 2020 (2020). Available online at: http://www.governo.it/it/articolo/firmato-il-dpcm-9-marzo-2020/14276 (accessed May 22, 2020).

[4] Garattini L Zanetti M-Freemantle N. The Italian NHS: what lessons to draw from COVID-19? Appl Health Econ Health Policy. (2020) 18:463–6. 10.1007/s40258-020-00594-5 32451979 PMC7247917

[5] LiBassi L Hwenda L. COVID-19: time to plan for prompt universal access to diagnostics and treatments. Lancet Global Health. (2020) 8:e756–7. 10.1016/S2214-109X(20)30137-6 32305076 PMC7162629

[6] IMF World Economic Outlook: The Great Lockdown (2020). Washington, DC Available at: https://www.imf.org/en/Publications/WEO (accessed May 22, 2020).

[7] Maffettone P Oldani C. COVID-19: a make or break moment for global policy making. Global Policy. (2020) 11:501–7. 10.1111/1758-5899.12860 32904957 PMC7460972

[8] Vanni G Materazzo M Pellicciaro M Ingallinella S Rho M Santori F. Breast cancer and COVID-19: the effect of fear on patients' decision-making process. In Vivo. (2020) 34:1651–9. 10.21873/invivo.11957 32503825 PMC8378027

[9] Vanni G Pellicciaro M Materazzo M Palombi M Buonomo OC. Breast cancer diagnosis in COVID19-Era: alert from Italy. Front Oncol. (2020) 10:938. 10.3389/fonc.2020.00938 32574281 PMC7258188

[10] World Health Organization Early Diagnosis and Screening: Breast Cancer. (2020) Available online at: https://www.who.int/cancer/prevention/diagnosis-screening/breast-cancer/en/ (accessed May 22, 2020).

[11] Eurostat Cancer Statistics (2019) Available online at: https://ec.europa.eu/eurostat/statistics-explained/index.php/Cancer_statistics_-_specific_cancers#Breast_cancer (accessed May 22, 2020).

[12] Foca F Mancini S Bucchi L Puliti D Zappa M Naldoni C. Decreasing incidence of late-stage breast cancer after the introduction of organized mammography screening in Italy. Cancer. (2013) 119:2022–8. 10.1002/cncr.28014 23504860

[13] Caplan L. Delay in breast cancer: implications for stage at diagnosis and survival. Front Public Health. (2014) 2:87. 10.3389/fpubh.2014.00087 25121080 PMC4114209

[14] Burrell HC Pinder SE Wilson ARM Evans AJ Yeoman LJ Elston CW. The positive predictive value of mammographic signs: a review of 425 non-palpable breast lesions. Clin Radiol. (1996) 51:277–81. 10.1016/S0009-9260(96)80346-1 8617041

[15] Ritchie D VanHal G VanDenBroucke S. How is informed decision-making about breast cancer screening addressed in Europe? An international survey of 28 countries. Health Policy. (2020) 124:1017–31. 10.1016/j.healthpol.2020.05.011 32709368

[16] Cedolini C Bertozzi S Londero AP Bernardi S Seriau L Concina S. Type of breast cancer diagnosis, screening, and survival. Clin Breast Cancer. (2014) 14:235–40. 10.1016/j.clbc.2014.02.004 24703317

[17] Osservatorio Screening Lo Screening Mammografico (2018). Available online at: https://www.osservatorionazionalescreening.it/content/lo-screening-mammografico (accessed May 22, 2020).

[18] Wübker A. Explaining variations in breast cancer screening across European countries. Europ J Health Econ. (2014) 15:497–514. 10.1007/s10198-013-0490-3 23744174

[19] Goldzahl L. Contributions of risk preference, time orientation and perceptions to breast cancer screening regularity. Soc Sci Med. (2017) 185:147–57. 10.1016/j.socscimed.2017.04.037 28578212

[20] Mongey S Pilossophz L Weinbergx A Which Workers Bear the Burden of Social Distancing Policies? (2020). Available online at: https://www.nber.org/papers/w27085 (accessed May 22, 2020). 10.3386/w27085 PMC8328128 34366750

[21] OECD The Pursuit of Gender Equality: An Uphill Battle. How Does Italy Compare? (2017). Available online at: http://www.oecd.org/gender/the-pursuit-of-gender-equality-9789264281318-en.htm (accessed May 22, 2020).

[22] Bleicher RJ. Timing and delays in breast cancer evaluation and treatment. Ann Surg Oncol. (2018) 25:2829–39. 10.1245/s10434-018-6615-2 29968031 PMC6123282

[23] Senie RT Lesser M Kinnie DW Rosen PP. Method of tumor detection influences disease-free survival of women with breast carcinoma. Cancer. (1994) 73:1666–72. 10.1002/1097-0142(19940315)73:6<1666::AID-CNCR2820730619>3.0.CO;2-E 8156494

[24] Buonomo OC Materazzo M Pellicciaro M Caspi J Piccione E Vanni G. Tor Vergata University-Hospital in the beginning of COVID-19-Era: experience and recommendation for breast cancer patients. In Vivo. (2020) 34:1661–5. 10.21873/invivo.11958 32503826 PMC8378037

[25] Blumen H Fitch K Polkus V. Comparison of treatment costs for breast cancer, by tumor stage and type of service. Am Health Drug Benefits. (2016) 9:23–32. 27066193 PMC4822976

[26] Reeves A. The EU and the social determinants of health in a post-COVID world. Eur J Public Health. (2020) 30:625–6. 10.1093/eurpub/ckaa100 32639000

[27] Eurogroup. Report on the Comprehensive Economic Policy Response to the COVID-19 Pandemic, 9 April (2020) (accessed May 22, 2020).

[28] European Commission Public Health. (2020) Available online at: https://ec.europa.eu/info/live-work-travel-eu/health/coronavirus-response/public-health_en (accessed June 22, 2020).

[29] Vanni G Materazzo M Santori F Pellicciaro M Caspi J Buonomo OC. The effect of coronavirus (COVID-19) on breast cancer teamwork: a multicentric survey. In Vivo. (2020) 34:1685–94. 10.21873/invivo.11962 32503830 PMC8378028

